# Language and sensory characteristics are reflected in voice-evoked responses in low birth weight children

**DOI:** 10.1038/s41390-024-03270-9

**Published:** 2024-06-21

**Authors:** Yuko Yoshimura, Yusuke Mitani, Takashi Ikeda, Sanae Tanaka, Momoka Suda, Ken Yaoi, Chiaki Hasegawa, Kyung-min An, Sumie Iwasaki, Hirokazu Kumazaki, Daisuke N. Saito, Hidenobu Ohta, Akiko Ando, Kazutoshi Cho, Mitsuru Kikuchi, Taizo Wada

**Affiliations:** 1 Institute of Human and Social Sciences, Kanazawa University, Kakuma-machi, Kanazawa, 920-1192 Japan; 2 Research Center for Child Mental Development, Kanazawa University, 13-1 Takara-machi, Kanazawa, 920-8640 Japan; 3 Department of Pediatrics, Kanazawa University, 13-1 Takara-machi, Kanazawa, 920-8640 Japan; 4 Research Center for Child Mental Development, Hamamatsu University School of Medicine, 1-20-1 Handayama, Higashi-ku, Hamamatsu, Shizuoka 431-3192 Japan; 5 Department of Psychology, Teikyo University, 2-11-1 Kaga, Itabashi-ku, Tokyo, 173-8605 Japan; 6 Centre for Human Brain Health, School of Psychology, University of Birmingham, Birmingham, B15 2TT UK; 7 Department of Future Psychiatric Medicine, Graduate School of Biomedical Sciences, Nagasaki University, 1-12-4 Sakamoto, Nagasaki, 852-8521 Japan; 8 Department of Psychology, Yasuda Women’s University, 6-13-1 Kuyasu, Asaminami, Hiroshima 731-0153 Japan; 9 Department of Occupational Therapy, Akita University Graduate School of Medicine, 1-1-1 Hondo, Akita, 010-8543 Japan; 10 Maternity and Perinatal Care Center, Hokkaido University Hospital, N15, W7, Kita-Ku, Sapporo, 060-8638 Japan; 11 Department of Psychiatry and Neurobiology, Graduate School of Medical Science, Kanazawa University, 13-1 Takara-machi, Kanazawa, 920-8641 Japan

## Abstract

**Background:**

Children born with very low birth weight (VLBW) are at higher risk for cognitive impairment, including language deficits and sensorimotor difficulties. Voice-evoked response (P1m), which has been suggested as a language development biomarker in young children, remains unexplored for its efficacy in VLBW children. Furthermore, the relation between P1m and sensory difficulties in VLBW children remains unclear.

**Methods:**

40 children with VLBW were recruited at 5-to-6 years old (26 male, 14 female, mean age of months ± SD, 80.0 ± 4.9). We measured their voice-evoked brain response using child-customized magnetoencephalography (MEG) and examined the relation between P1m and language conceptual inference ability and sensory characteristics.

**Results:**

The final sample comprised 36 children (23 boys, 13 girls; ages 61–86 months; gestational ages 24–36 weeks). As a result of multiple regression analysis, voice-evoked P1m in the left hemisphere was correlated significantly with language ability (*β* = 0.414 *P* = 0.015) and sensory hypersensitivity (*β* = 0.471 *P* = 0.005).

**Conclusion:**

Our findings indicate that the relation between P1m and language conceptual inference ability observed in term children in earlier studies is replicated in VLBW children, and suggests P1m intensity as a biomarker of sensory sensitivity characteristics.

**Impact:**

- We investigated brain functions related to language development and sensory problems in very low birth-weight children.
- In very low birth weight children at early school age, brain responses to human voices are associated with language conceptual inference ability and sensory hypersensitivity.
- These findings promote a physiological understanding of both language development and sensory characteristics in very low birth weight children.

## Introduction

Language ability is a key factor for successful communication and social interaction and for educational achievement. Very low birth weight (VLBW), defined as a birth weight of less than 1500 g, is associated with more difficulties with language ability at school age.^1^ Numerous studies have specifically undertaken elucidation of the physiological mechanisms underlying child language development. Particularly, in children aged 1 to 10 years, the auditory evoked P1(m) is a prominent component in both hemispheres^2–6^ providing insight into the development of auditory processing. In studies using magnetoencephalography (MEG), the mid-latency auditory evoked field (AEF) comprises the P50 m (P1m), N100 m and P200m components. Of them, P1m is a mid-latency component corresponding to the P50 (P1) in electroencephalography (EEG) studies^2,5^ and is associated with the processing of human voices. Our earlier reports have described the P1m component as related to human voice processing and as associated with age,^7^ intelligence^8^, and language conceptual inference ability^9^ in neurotypical full-term birth children. Other studies investigating the relationship between P1m and developmental disorders have suggested that voice-evoked P1m intensity could serve as a biomarker for language development in young children.^10^ Nevertheless, whether the P1m response intensity evoked by human voice can be a biomarker for language development in preterm and low birth weight children, who face high risk of neurodevelopmental disorders, has not been reported in the relevant literature.

Altered sensory processing is also commonly reported in children born with VLBW.^11^ Children with VLBW are at high risk for the development of sensorimotor difficulties, including those related with auditory, visual, tactile, postural stability, and kinesthetic processing^12–14^ and display greater hyper-sensory and hypo-sensitive behaviors than children born at full-term.^15–17^ A prior study has indicated a link between sensory issues in children with low birth weight and severe cognitive and language difficulties.^17^ Given the numerous reports describing the relationship between language and sensorimotor functions,^18–21^ these findings suggest a common neural basis associated with these symptoms.

The previously described auditory evoked P1m, a physiological index of language development, has also been suggested to be associated with auditory hypersensitivity in children with autism spectrum disorder (ASD).^22^ On the other hand, it is not known whether this relationship is also observed in VLBW and preterm children.

However, it is hypothesized that preterm and VLBW children, who are at high risk for developmental disorders and have significant language and sensory issues, may also exhibit similar characteristics in cortical activity to those with ASD.

Therefore, this study specifically focuses on the relationship between P1m and language and sensory characteristics to elucidate the physiological background related to language development and sensory difficulties in children with VLBW. We hypothesized that the intensity of P1m is associated with language conceptual inference abilities and sensory difficulties in children with VLBW.

## Methods

### Participants

From the university hospital, 40 children with low birth weight were recruited at 5 to 6 years old (26 male, 14 female; mean age of month ± SD, 80.0 ± 4.9). The inclusion criteria for participants with very low weight birth in this study were the following: (1) preterm birth (at less than 36 weeks’ gestational age and having a birth weight of less than 1500 g (very low birth weight)) and (2) absence of chromosomal or other major genetic abnormalities, absence of suspected neuromuscular disorders, absence of grade 3 or higher intraventricular hemorrhage (IVH), and absence of significant periventricular leukomalacia (PVL), cerebral malformations and chronic lung disease (CLD). We used the Autism Diagnosis Observation Schedule (ADOS-2)^23^ to evaluate the characteristics of autism spectrum disorder (ASD). Cognitive skills were assessed using the Japanese translation of the Kaufman Assessment Battery for Children (K-ABC).^24^ Language conceptual inference ability was measured using the riddle task in K-ABC. In the riddle task, children must retrieve a target word according to suggested words that are associated conceptually with the target word. The parents agreed to the participation of their child in the study with full knowledge of the experimental characteristics of the research. Written informed consent was obtained from each participant before the study. The Ethics Committee of Kanazawa University Hospital approved the methods and procedures, all of which were used in accordance with the Declaration of Helsinki.

### Sensory assessment

The Sensory Profile (SP) is a standardized questionnaire with behavioral statements in which caregivers rate their children’s responses to sensory events that occur in daily life.^25^ The Caregiver Sensory Profile was published in its Japanese version in 2015.^26^ This questionnaire includes 125 questions that quantify the frequency of abnormal behavioral responses to various sensory experiences.^25^ The parent or caregiver rates how frequently the child responds to regularly occurring sensory events in a particular manner using a Likert scale with responses ranging from 1 (never) to 5 (always). The SP includes four domains: Low Registration/ Sensation Seeking/ Sensory Sensitivity/and Sensation avoiding. In this study, standardized scores for these four domains were used for analysis.

### Auditory-evoked field stimuli and procedures

Magnetoencephalography (MEG) recordings were obtained from all participants during auditory syllable sound stimulation consisting of the Japanese syllable /ne/.^9^ We used typical oddball sequences consisting of standard stimuli (456 times, 84%) and deviant stimuli (90 times, 16%). In the standard stimulus, /ne/ was pronounced with a steady pitch contour, but in the deviant condition /ne/ had a falling pitch. Eventually, we adopted only standard stimuli for analysis in this study. A female native Japanese speaker produced the /ne/ sounds, which were recorded using a condenser microphone (NT1-A; Rode, Silverwater, NSW, Australia) and a personal computer. The stimulus duration was 342 ms; the duration of the consonant/n/ was 65 ms. The interstimulus interval (ISI) was 818 ms. Each stimulus had an intensity level of approximately 65 dB (A-weighted) at the head position against a background noise level of 43 dB. Intensity was measured using an integrating sound level meter (LY20; Yokogawa Electric Corp.). The stimulus was presented to the participants binaurally through tubes fixed to the dewar. The recording was 12 min long.

### Magnetoencephalography recordings

The auditory cortical responses were recorded using a 151-channel Superconducting Quantum Interference Device (SQUID) whole-head coaxial gradiometer MEG system for children (PQ 1151 R; Yokogawa/KIT, Kanazawa, Japan) in a magnetically shielded room (Daido Steel Co. Ltd., Nagoya, Japan) installed at the MEG Center of Ricoh Co. Ltd. (Kanazawa, Japan). One researcher remained in the room to encourage the children and to prevent them from moving during the analysis. The head location relative to the MEG device helmet was measured using four coils attached to the head surface as fiduciary points with respect to the landmarks (bilateral preauricular points, Cz, and 5 cm anterior part from Cz) for children. Before the MEG session, a three-dimensional digitizer (FASTRAK; Polhemus Inc., Colchester, VT) was used to digitize the head surface points and fiduciary landmarks of the participant. After MEG recording, the positioning coils were replaced with MRI-visible markers.

### MRI recording

Brain structural images were obtained individually for source confirmation from participants, except for 8 children using a 1.5 T MRI scanner (SIGNA Explorer; GE Healthcare, Chicago, IL). For each participant, an MRI scan was acquired using the T1-weighted gradient echo and Silenz pulse sequence (TR = 435.68 ms, TE = 0.024 ms, 7° flip angle, 220 mm FOV, 256 × 256 pixel matrix size, 1.7 mm slice thickness, and 130 transaxial images). A spherical model was used for source analysis for the 8 children who did not complete MRI imaging.

### Data analysis

The band pass-filtered MEG data (0.16–200 Hz) were collected at a sampling rate of 2000 Hz. The time series obtained started from the onset of the syllable stimulus at -100 ms and continued to 900 ms. Subsequent segments were averaged for each sensor after baseline correction (0–50 ms). Segments that were contaminated with artifacts (eye-blink, eye and body movements, typically more than ± 4 pT) were excluded from the analysis. A single ECD model was used to estimate the current sources in the activated cerebral cortex using more than 40 sensors for each hemisphere (left and right). To estimate the localization of the current sources, MegLaboratory 160 (Yokogawa/KIT, Kanazawa, Japan) was used. To identify the P1m component, we accepted estimated ECDs when the following were found: (1) the goodness of fit (GOF) exceeded 80%; (2) the location of the estimated dipoles using a single ECD model was stabilized to ± 5 mm of each coordinate for at least 6 ms during the target response period; (3) the dipole intensities were ≤ 80 nAm; and (4) the direction of the estimated ECD was in an anterosuperior orientation. The latency time point was defined as the maximum estimated dipole intensity value obtained in accordance with the above criteria within a time window of 40–150 ms.

### Statistical analysis

Statistical analyses were conducted using software (Statistical Package for the Social Sciences (SPSS) for Windows, ver. 25.0; IBM Japan Ltd., Tokyo, Japan). A paired *t*-test was used to investigate differences in P1m intensity between the left and right hemispheres (*P* < 0.05). To assess the relation between language ability and the P1m intensity in both hemispheres, Pearson’s correlation coefficient was used (*P* < 0.05/2 = 0.025). For the relation between P1m intensity in both hemispheres and sensory characteristics, Pearson’s correlation coefficient was used (*P* < 0.05/4 = 0.012). Furthermore, when a significant relation between P1m intensity and language ability and/or sensory characteristics was found, a multiple regression analysis was performed, which included language and sensory factors and other factors that might affect relations such as age, gestational weeks, birth weight and variables for meeting or not meeting diagnostic criteria for ASD using ADOS (*P* < 0.05). Our earlier research has shown that the voice-evoked P1m intensity in the left hemisphere is related to language conceptual inference ability in neurotypically developing children, but this relation is not observed in children with ASD.^27^ Therefore, whether or not ASD criteria were met might affect the relation between P1m strength and language conceptual inference ability. For that reason, it was used as an independent variable for multiple regression analysis. As a complementary analysis, we examined the relation between P1m intensity and language and sensory factors separately for male and female children using Pearson’s correlation coefficient. The statistical significance level was set at *P* < 0.025 for language ability and *P* < 0.012 for sensory characteristics.

## Results

Of the 40 children recruited, 36 children met the inclusion criteria and completed the study. Of the four participants who were unable to complete the study, two children were unable to perform the K-ABC cognitive assessment, and two children were unable to provide MEG measurements. The average age of the 36 children analyzed (23 boys and 13 girls) was 70.7 mean months (61–86 months). Of the 36 preterm children, 7 (19.4%) met the criteria for ASD as assessed by ADOS. None of the children exceeded the cutoff with a 90 percentile value or higher when ADHD characteristics were assessed using the ADHD-Rating Scale.^28^ The presence or absence of complications associated with the Neonatal Intensive Care Unit (NICU), such as grade 2 or lower IVH, periventricular leukomalacia (PVL), or necrotizing enterocolitis (NEC), was confirmed in the participants. Among the participants, two children were found to have grade 2 or lower IVH. Furthermore, one child did not have PVL on MRI during hospitalization, but the prognosis was mild PVL. The other patient did not have NEC but had a mesenteric hernia and underwent colostomy management during hospitalization. Therefore, these two cases were further included in those exhibiting complications associated with the NICU. The participant characteristics are presented in Table 1. As a supplementary material, the causes of preterm birth among participants, maternal pregnancy complications, and the duration of hospital stay (days) are presented in the Supplementary Table.

**Table 1:** Characteristics of participants.

	*n* = 36
Sex (male/female)	23/13
Chronological age (months) (median, IQR)	70(7)
Birth weight (g) (median, IQR)	1096 (722-1287)
Gestational age at birth (weeks) (median, IQR)	28.5 (27-30.75)
Apgar score at birth (5 min) (median, IQR)	9 (8-9)
Complications related to the NICU (yes/no)	4/32
K-ABC	
Mental processing scale (mean,SD)	92.0 (14.7)
Language conceptual inference task (Riddles) (mean,SD)	91.5 (17.3)
Sensory profile	
Low registration (mean,SD)	22.8 (7.3)
Sensory seeking (mean,SD)	34.9 (7.8)
Sensory sensitivity (mean,SD)	28.8 (4.0)
Sensation avoiding (mean,SD)	45.5 (8.3)

K-ABC, Kaufman Assessment Battery for Children; NICU, Neonatal Intensive Care Unit.

Sex was confirmed by medical records at birth and documents containing information about the child completed by parents at the time of study participation.

### P1m intensity in very low birth weight children

**Fig. 1:**
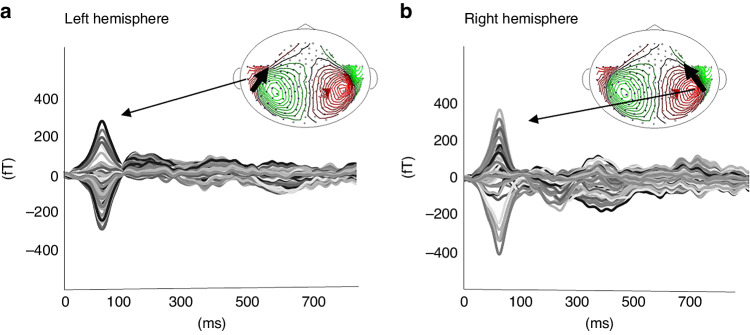
Source modeling of the P1m response evoked by voice from a 5-year-old female with low birth weight. AEF waveform and dipole location of P1m response in both Heschl’s gyri in a child. The P1m response showed an activity peak at 40-150 ms in the left hemisphere (**a**) and the right hemisphere (**b**). Black arrows indicate the directions of equivalent current dipole orientation.

For 36 VLBW children, the P1m component which met the detection criteria was obtained in 30 left hemispheres and 31 right hemispheres. The P1m dipole intensities were 23.3 ± 11.0 and 17.7 ± 7.5 nAm (mean ± SD), respectively, in the left and right hemispheres. Paired *t*-test results indicated the P1m intensity in the left hemisphere as significantly higher than in the right hemisphere (*n* = 27, *t* = 2.475, *P* = 0.020). Source modeling of the P1m response evoked by voice in the left and right hemispheres is shown in Fig. 1.

### Relation between P1m and language performance

**Fig. 2:**
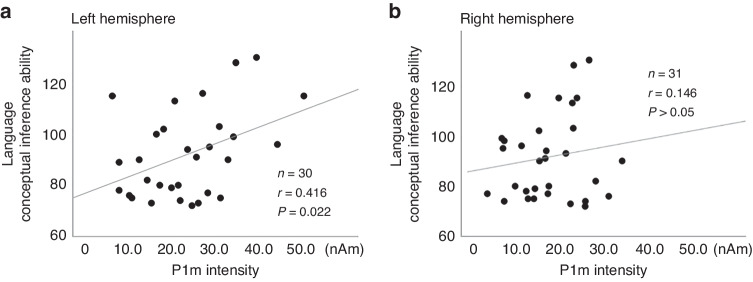
Scatter plot of P1m intensity and language performance. P1m responses showed a significant correlation with language conceptual inference ability in the left hemisphere (**A**), but not in the right hemisphere (**B**).

By specifically examining language ability which was found to be associated significantly with P1m intensity in earlier research, we investigated the relation between language conceptual inference ability and P1m intensity using Pearson’s correlation coefficient. A significant correlation was found between P1m intensity in the left hemisphere and language conceptual inference ability (i.e. riddles) (*n* = 30, *r* = 0.416, *P* = 0.022, Fig. 2). No significant relation with P1m intensity was found in the right hemisphere. As a supplementary analysis, when we examined the relation between P1m intensity and language performance separately for male and female children, the results indicated no significance in either hemisphere for either group.

### Relation between P1m and sensory characteristics

**Fig. 3:**
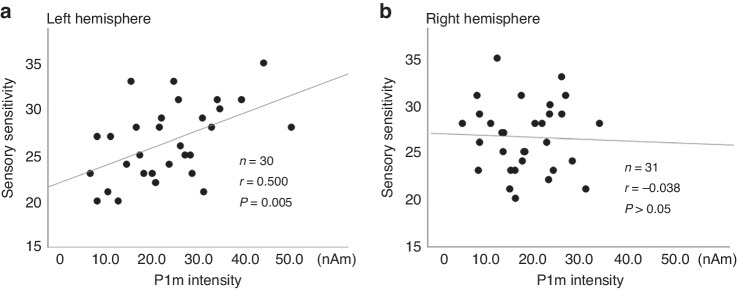
Scatter plot of P1m intensity and sensory characteristics. P1m responses showed a significant correlation with hypersensitivity in the left hemisphere (**A**), but not in the right hemisphere (**B**).

**Fig. 4:**
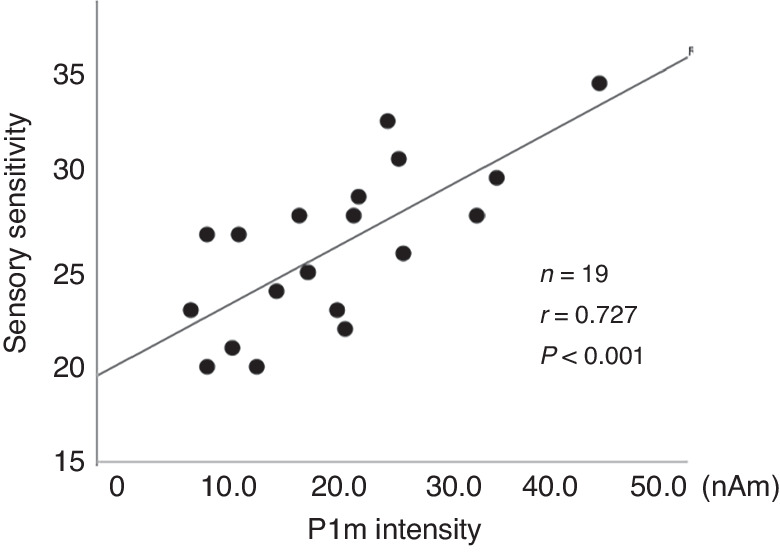
Scatter plot of P1m intensity in the left hemisphere and sensory characteristics in male children. P1m responses showed a significant correlation with sensory hypersensitivity in the left hamisphere in males (*n* = 19).

For VLBW children, significant correlation between P1m intensity in the left hemisphere and sensory sensitivity in SP (*n* = 30, *r* = 0.500, *P* = 0.005, Fig. 3), but not the right hemisphere. The remaining three domains (i.e. low registration/sensory seeking/sensation avoiding) were not significantly correlated with P1m intensity for either hemisphere. As a supplementary analysis, when we investigated the relation between P1m intensity and sensory hypersensitivity separately for male and female children, a significant correlation was found between the left P1m intensity and sensory hypersensitivity in male children (*r* = 0.727 *P* < 0.001, Fig. 4).

### Relation between P1m and language ability and sensory characteristics

To elucidate the relation between left P1m intensity and language and/or sensory difficulties, and to consider factors that may influence those relationships, multiple regression analysis was performed with the left P1m intensity as the dependent variable, and with the age, variables for a meeting or not meeting diagnostic criteria for ASD, gestational age, birth weight and language conceptual inference ability and sensory sensitivity as independent variables. Results show left P1m intensity as related significantly to the language conceptual inference ability (Table 2: *F* = 4.671, *P* < 0.05; riddle; *β* = 0.414 *P* = 0.015) and sensory sensitivity (*β* = 0.471 *P* = 0.005). Furthermore, to investigate the relation between left P1m intensity and sensory hypersensitivity specifically among male children, we conducted a multiple regression analysis. In this analysis, we included independent variables such as age in months, presence or absence of ASD characteristics, gestational weeks, birth weight, language abilities, and sensory hypersensitivity indicators. Results show that only sensory hypersensitivity was significantly correlated with P1m intensity in the left hemisphere (*n* = 19, *F* = 3.851 *P* = 0.022; sensory sensitivity, *β* = 0.711, *P* = 0.002).

**Table 2:** Left P1m intensity and language performance or sensory sensitivity.

	β						
	Step 1	Step 2	Step 3	Step 4	Step 5	Step 6	*t* in step 6
Age in months	−0.046	−0.053	−0.054	−0.093	0.010	−0.019	−0.108
Presence or absence of ASD characteristics		0.105	0.098	0.107	0.233	0.198	1.164
Gestational week			−0.029	0.092	0.24-	0.046	0.176
Birth weight				−0.164	0.191	−0.068	−0.272
Language conceptual inference ability (K-ABC)					0.494	0.415	2.401*
Sensory sensitivity (SP)						0.465	2.815**
F	0.059	0.179	0.122	0.160	1.457	2.887	
*R*	0.046	0.114	0.118	0.158	0.483	0.655	
Adjusted *R*^2^	−0.034	−0.060	−0.100	−0.131	0.073	0.281	
Δ*R*^2^	0.002	0.011	0.001	0.011	0.208	0.197	

^*^P < 0.05, **P < 0.0125. *K-ABC* Kaufman Assessment Battery for Children, *SP* sensory profile.

## Discussion

This study specifically addressed VLBW children and examined the relation between the P1m response in the auditory cortex, evoked by human voices, and language and sensory characteristics. Results of this study revealed that, in VLBW children, the intensity of P1m in the left hemisphere to human voices is associated with both language and sensory hypersensitivity characteristics.

This report is the first to describe a study assessing brain responses to human voices using child-customized MEG in preterm and VLBW children at early school age. In preterm and VLBW children, voice-evoked P1m intensity in the left hemisphere was found to be significantly higher than in the right hemisphere. This leftward lateralization of the neurophysiological responses to speech stimuli is consistent with findings from our earlier study examining term children^14^ and findings presented in several earlier reports.^29,30^ Our results provide further evidence of physiological features in VLBW children during conscious conditions.

Our findings indicate that voice-evoked brain response in the left hemisphere is associated with language ability. Earlier reports have described brain leftward lateralization as an intriguing property of human brain development that is associated with language acquisition.^9,31–34^ Using EEG, Kuuluvainen et al.^34^ showed that larger P1 in response to speech sounds on the left than on the right were related to better phonological and prereading skills in 6–7-year-old children. Using a MEG system that is capable of independently estimating dipole sources in the left and right brain hemispheres, we obtained additional neurophysiological findings that are consistent with these earlier EEG studies.^35,36^ Our results revealed that stronger P1m intensity in response to speech sounds in the left hemisphere is related to better language conceptual inference ability in VLBW children. This relation suggests that maturation of the nervous system related to speech processing in VLBW children is related to the neural substrates involved in the language conceptual inference ability, as in term children. Although this relation between P1m intensity in the left hemisphere and language ability was not observed in ASD children in our earlier studies,^27,37^ VLBW children reproduced the same relation as that found for children born at full term.

In addition, our results demonstrated an association between P1m intensity in the left hemisphere and features of sensory hypersensitivity in VLBW children. Although VLBW children participating in this study showed no apparent symptoms of hypersensitivity in the SP scores, our findings indicate a larger brain cortical response to auditory stimuli as associated with sensory hypersensitivity, which is consistent with findings presented in other reports describing studies of children with ASD and hypersensitivity. Few researchers have examined sensory issues and physiological indicators in children. Matsuzaki^38^ reported that the tone-evoked P1m intensity in ASD children with hypersensitivity was greater than those in TD or ASD children without hypersensitivity.^38^ Donkers et al.^39^ also reported that the combination of P1 and subsequent auditory components to auditory stimuli using an EEG is associated with sensory characteristics (i.e., hyperresponsiveness, hyporesponsiveness, and sensory seeking) in 4 − 12-year-old children with ASD.^39^ Furthermore, ASD children with atypical sensory behavior showed increased cortical activation to integrated visual and auditory stimuli in visual, auditory areas, and insula than TD children and ASD children without atypical sensory behavior.^40^ Furthermore, several reports have described that multiple regions of the brain are activated by certain stimulation modalities.^41,42^ Using functional Magnetic Resonance Imaging (fMRI), Acevedo et al.^42^ investigated the brain activity of adult men with a high score of highly sensitive personality (HSP) while observing various facial expressions of their loved ones and strangers. Their results indicated that HSP scores are associated with increased brain activation of regions involved in attention and action planning (in the cingulate and premotor area).^42^ Our results reinforce the findings of earlier necessary studies showing a link between sensory hypersensitivity and excessive brain activation in VLBW children. Low birth weight children who are adversely affected by sensory difficulties might be characterized by brain function that is susceptible to, or hyperactive in response to stimuli, similar to children with ASD. Therefore, it is possible that the magnitude of the brain response obtained in the auditory cortex to speech, which is a social stimulus in this study, reflects the activation of multiple regions, including the auditory cortex in the whole brain. It is also necessary to perform whole-brain analyses such as current source estimation and network analysis.

Based on the results of our earlier study,^27^ we considered the possibility that whether a child met the criteria for ASD might influence the relation between the intensity of P1m and language conceptual inference ability. Therefore, we used it as an independent variable for multiple regression analysis. However, in the analysis applied for this study, results indicated that the intensity of P1m in the left hemisphere and its relation to language and sensory functions were unaffected by whether the child met the criteria for ASD. Possible factors contributing to this result might include the limited number of children matching the diagnosis of ASD and potential differences in language development and sensory function maturation between VLBW children with ASD and full-term children with ASD. Although no definitive conclusions related to this matter can be drawn from the results of this study, we hope to expand the sample of individuals with ASD and to explore this matter further in future research.

It is intriguing that some physiological studies have demonstrated a link between sensory processing, cognitive function, and the insular cortex.^43–46^ The insular cortex, a region that controls interoceptive sensations, efficiently organizes bottom-up body signals (inputs) from peripheral organs such as body parts and internal organs, transmitting information to other brain regions, and also receiving and controlling (input) from other brain regions.^46–48^ Based on findings indicating that insula is involved in language ability^49^ and considering results showing that altered sensory integration is associated with language development in individuals with ASD,^50^ the insula might be a common region affecting both language and sensory function. Earlier structural reports have described altered gray matter volume and atypical activation^51,52^ of the insula in very low birth weight individuals. Although it is a speculative consideration in light of the correlations found from this study, dysfunction of the insular cortex in VLBW children might affect sensory sensitivity and language acquisition, which might be reflected in the magnitude of the auditory response (i.e. P1m). Additionally, in terms of the recent concept of whole-brain network analysis, the insular cortex plays a central role in information transmission in the brain. It is therefore involved in the formation of a rich club. In fact, weakened rich-club connectivity has been reported in the brains of preterm children^53,54^ and children with developmental disorders.^55,56^ In VLBW children, cerebral white matter disorders have been reported as a typical abnormality.^57,58^ For that reason, it is reasonable to infer that the networks in the brain itself will be fewer. Furthermore, the influence might appear in the intensity of the response to voice stimuli (i.e. P1m). Several reports have described that the insular cortex is involved in the processing of sound stimuli,^59–61^ which also supports the suggestion in this study that the function of the insular cortex is also reflected in the amplitude of the auditory response (P1m).

## Limitations

Our current neurofunctional research specifically examines children in early childhood who demonstrate vulnerability in brain development because of preterm and low birth weight. By accurately capturing the responses of the auditory cortices in both hemispheres, this study contributes to further elucidation and confirmation of the development of neural systems related to children’s language development and sensory characteristics. However, this study has some general limitations. Due to the small number of participants and insufficient statistical power, we may have failed to demonstrate significant relationships with other factors. First, we could not demonstrate that the intensity of P1m is related to gestational age and birth weight through a multiple regression model. Furthermore, we could not demonstrate that the intensity of P1m in the left hemisphere is influenced by whether the child had been diagnosed with ASD. Although no definitive conclusions related to this matter can be drawn from the results of this study, we hope to expand the sample of individuals matching ASD and to explore this matter further in future research. Additionally, maternal education level might influence preterm birth^62^; but it is noteworthy that maternal education was not assessed in this study. Furthermore, we investigated P1m using only one type of auditory stimulus: The human voice /ne/. Therefore, we are unable to determine whether our results are specific to human voice stimuli. Finally, we included only preterm and low birth weight participants in the study to account better for the known clinical heterogeneity of very low birth weight children. Therefore, we acknowledge that we have been unable to ascertain whether the observed findings are significantly different from those of children carried to term.

## Conclusion

At 5–6 years of age, children with VLBW were found to have significantly stronger P1m response in the left hemisphere to voice stimuli compared to the right hemisphere. A significant positive correlation was found between voice-evoked P1m intensity in the left hemisphere and language conceptual inference ability. We also found that the larger P1m intensity in the left hemisphere is associated with high sensory hypersensitivity characteristics. These findings suggest that the developmental process of the auditory cortex related to language ability in VLBW children is similar to that of neurotypical term children, but the possibility of malfunction exists in the neural mechanisms involved in sensory processing and information integration. Further neurological insights are necessary to elucidate the mechanism behind the integration of language ability and sensory sensitivity in children.

## Supplementary information

Supplementary Table

## Data Availability

The datasets generated during and/or analyzed during the current study are available from the corresponding author upon reasonable request.
